# Differences in the viral genome between HPV-positive cervical and oropharyngeal cancer

**DOI:** 10.1371/journal.pone.0203403

**Published:** 2018-08-30

**Authors:** Bailey A. LeConte, Peter Szaniszlo, Susan M. Fennewald, Dianne I. Lou, Suimin Qiu, Nai-Wei Chen, John H. Lee, Vicente A. Resto

**Affiliations:** 1 Department of Otolaryngology, The University of Texas Medical Branch at Galveston, Galveston, Texas, United States of America; 2 Department of Pathology, The University of Texas Medical Branch at Galveston, Galveston, Texas, United States of America; 3 Department of Preventive Medicine & Community Health, The University of Texas Medical Branch at Galveston, Galveston, Texas, United States of America; 4 Department of Adult Medical Affairs, Chan Soon-Shiong Institute of Molecular Medicine, Culver City, California, United States of America; Istituto Nazionale Tumori IRCCS Fondazione Pascale, ITALY

## Abstract

Human papillomavirus (HPV)-driven oropharyngeal cancer incidence in the United States has steadily increased in the past decades and has now become the most frequently diagnosed HPV-associated cancer type, surpassing cervical cancer. Variations in the HPV genome correlate with tumorigenic risk, and the distribution of genetic variants is extensively studied in cervical cancer, but very little is known about new mutations or the distribution of HPV types and variants in oropharyngeal cancer. Here we present an archival tissue cohort study that compares genomic characteristics of HPV associated with cervical versus oropharyngeal tumors using DNA sequence analysis. We found HPV16 to be more prevalent in oropharyngeal samples than in cervical samples (91.2% versus 52.9%), while HPV18 (1.5% versus 18.2%) and HPV45 (0.7% versus 9.9%) were much less prevalent. Differences between cervix and oropharynx in HPV16 variants distribution were more subtle, but the combined European + Asian (EUR+AS) variant group was more prevalent (90.2% versus 71.4%), while the American Asian 1 + American Asian 2 (AA1+AA2) variant group was much less prevalent (4.4% versus 22.5%) in oropharyngeal cancers. HPV prevalence in oropharyngeal cancers showed an increasing trend from 60% in 2003 to 80% in 2016. We also identified over nine times more nonsynonymous mutations in the HPV E6 gene amplified from oropharyngeal samples, but for E7 the difference in mutation rates between the two anatomical locations was not significant. Overall, we showed that HPV genome in oropharyngeal cancer presents important differences when compared to cervical cancer and this may explain the distinct pathomechanisms and susceptibility to treatment of HPV-associated oropharyngeal cancer.

## Introduction

Human papillomavirus (HPV) has been identified as an etiological agent for several cancer types, being responsible for over 600,000 cases each year worldwide [1]. Cervical and oropharyngeal cancers are the two leading HPV-associated cancer types with virtually all cervical squamous cell carcinomas (CSCC) and 30–80% of all oropharyngeal squamous cell carcinomas (OSCC) being attributed to HPV [2–4]; with the higher end of this spectrum (70–80% of OSCC) found in the United States and Western Europe [4, 5]. Thirteen out of the 225 currently reported HPV types [6] have been classified as high risk based on their high oncogenic potential. HPV16 alone has been associated with 50–70% of cervical cancers [7] and over 90% of HPV-positive oropharyngeal tumors [8, 9].

The role of the two major viral oncoproteins, E6 and E7, in cancer pathomechanisms has been extensively demonstrated, and together they have been shown to be necessary but not sufficient for HPV-driven cellular transformation [10–12]. In fact, E6 and E7 are multifunctional proteins, which bind–among other host proteins–cellular tumor suppressor proteins p53 and Rb, respectively, dysregulating the cell cycle and leading to oncogenic transformation [10, 13].

Several studies have shown the importance of variations in the HPV genome for its oncogenic potential and reported variant-specific differences in tumorigenic risk [14–16]. Recent reports described genomic polymorphisms in the E6 gene of HPV16 and suggested potential roles in tumor biology for some of them [17–19]. Interestingly, virtually no data exist about E7 polymorphism-related alterations in the oncogenic potential of HPV16.

Beginning in the 1970s, HPV-associated OSCC incidence in the US has been steadily increasing, while non-HPV related OSCC incidence has declined [20, 21]. During the same timeframe, HPV-related CSCC incidence rates have decreased in the US and other high-resource countries due to the implementation of screening programs for cervical cancer [22, 23]. As a result of these opposing trends, the incidence of HPV-related OSCC has surpassed that of CSCC in the US with 16,500 versus 11,700 new cases per year; respectively, and similar trends have been reported from other high-income countries as well [2, 24]. A strong correlation between sexual practices, especially orogenital sexual contact and HPV-positive OSCC incidence has been demonstrated [25–28], but a simple mechanistic view of HPV transmission from one anatomical location to the other may not be sufficient to explain the opposite trends in the incidences of HPV-associated CSCC and OSCC. A possible explanation for the dichotomy between cervical and oropharyngeal cancer incidence is genetic drift in the HPV genome that leads to an increasing rate of carcinogenesis in the oropharynx.

Most of our current understanding about HPV-driven cancer pathogenesis has derived from cervical cancer studies and OSCC-specific data about these pathomechanisms are very limited. The purpose of this study is to address this gap of knowledge by comparing CSCC and OSCC tissues and exploring differences in HPV type and HPV16 variant distribution as well as genetic polymorphisms in the E6 and E7 genes of HPV16 in cervical versus oropharyngeal cancers diagnosed in a Southeast Texas community.

## Materials and methods

### Patients and samples

This study was an archival tissue cohort assessment. All tissue samples included in the study were obtained from The University of Texas Medical Branch (UTMB Health) in Galveston, Texas. All methods used in this study were approved by the Institutional Review Board (IRB) of UTMB Health; approval number 14–0386. Individual informed consent was not obtained as study specimens were deemed to be exempt from consent by the IRB since the de-identified, archival specimens were retrospectively collected, all patient data were de-identified and anonymized prior to analysis.

The first tissue group was comprised of OSCC tissues. The samples originated from patients diagnosed by histopathology with squamous cell carcinoma of the oropharynx, tonsils, or base of tongue between 2003 and 2016. Among 270 cases fitting these criteria, 226 formalin fixed paraffin embedded (FFPE) samples were available. The second group of samples was comprised of CSCC tissues. These samples derived from patients diagnosed by histopathology with CSCC between 2011 and 2015. A total of 154 FFPE samples fitting these criteria were available.

### DNA extraction and amplification

All samples were evaluated by a UTMB pathologist and regions containing tumor cells were marked. A hypodermic needle was used to remove a portion of the tissue containing tumor cells for analysis. DNA was extracted from all the samples using the QIAamp DNA FFPE Tissue Kit (50) (Qiagen) following the manufacturer’s instructions. We extracted DNA samples from 226 oropharyngeal and 154 cervical cancer tissues. To verify the presence of sufficient amount and quality of the extracted DNA, a PCR targeting a segment of the human CD273 gene was performed on each sample and samples that did not produce a visible band via agarose gel-electrophoresis were eliminated from the study. HPV DNA was detected by PCR followed by agarose gel-electrophoresis using the GP5+/GP6+ primer set amplifying a 140–150 base pair segment of the L1 gene under the published conditions [29]. E6- and LCR-specific primer sets were used to identify the HPV16 variants following established protocols [30]. To identify new mutations, six specific, overlapping pairs of primers were used for amplifying E6 sequences and 4 overlapping pairs of primers were used for E7 sequences in HPV16 positive samples. The following primer pairs were used for E6 amplification (5’-3’): E6-1F (CGAAACCGGTTAGTATAA) and E6-1R (GTATCTCCATGCATATT), E6-2F (TGTTTCAGGACCCACAGGAG) and E6-2R (AGGACACAGTGGCTTTTGACA), E6-3F (AAACTAAGGGCGTAACCGAAA) and E6-3R (TCCTCCTCCTCTGAGCTGTC), E6-4F (GATGGGAATCCATATGCTGTATGT) and E6-4R (CAGCTGGGTTTCTCTACGTG), E6-5F (AAGGCCAACTAAATGTCACCCTA) and E6-5R (TCACGTCGCAGTAACTGTTG), E6-6F (CAGGAGCGACCCAGAAAGTT) and E6-6R (TCCTCCTCCTCTGAGCTGTC). E7 primer pairs used in the study were (5’-3’): E7-1F (ACCGGTCGATGTATGTCTTG) and E7-1R (CCATCCATTACATCCCGTACCC), E7-2F (ACAACAAACCGTTGTGTGATTTG) and E7-2R (TTTGTACGCACAACCGAAGC), E7-3F (GACAGCTCAGAGGAGGAGGA) and E7-3R (TGAGAACAGATGGGGCACAC), E7-4F (GGAAGACCTGTTAATGGGCAC) and E7-4R (ATACCCGCTGTCTTCGCTTTC). All PCR reactions were carried out in 20 μL reaction volume using reagents from Phusion High-Fidelity DNA Polymerase kit (BioLabs) and BioLabs dNTP Solution Mix. Amplifications were performed in a Bio-Rad T100 Thermal Cycler. The following amplification protocol was used for all PCR reactions: First denaturation step at 98°C for 2 min followed by 35 cycles of 98°C for 10 s, 65°C for 30 s, 72°C for 1 min. This was followed by a final extension at 72°C for 5 min and then storage at 4°C. Samples that did not yield amplicons for any segment of the E6 and E7 genes were excluded from the corresponding mutation analysis part of the study.

### Sequence analysis

Unused primers and nucleotides were removed from the PCR products using ExoSAP-IT (Affymetrix) according to the manufacturer’s instructions. PCR products then underwent Sanger sequencing in the Sequencing Core Facility at UTMB Health. Bi-directional sequence detection was carried out on an Applied Biosystems 3130XL Genetic Analyzer followed by post-detection processing. As a result, a report consisting of raw data (Electropherogram), processed sequence, and quality scores (Phred) was produced. The obtained sequences were aligned to the reference HPV genomes of the NCBI database to identify the HPV type, HPV16 variant, and all mutations in the E6 and E7 genes present in the samples, using Geneious R8.1 (Biomatters) software. All HPV positive samples were included in HPV type analysis and all HPV16 positive samples were included in variant analysis, regardless of how complete E6 and E7 sequence data they produced. Contrarily, only samples with complete E6 or E7 sequence information were included in the corresponding part of the HPV16 mutation analysis. All mutations identified by this method were verified by an independent, second PCR and sequencing. 3D visualization of HPV E6 and E7 proteins was carried out using Cn3D 4.3.1 3-D structure viewer software (https://www.ncbi.nlm.nih.gov). We also evaluated the potential impact of each mutation on the structure and/or function of the protein based on the Sneath’s index (SI) of the exchanged amino acids [31].

### Statistical analysis

The data were summarized using frequencies and percentages for HPV types, variants of HPV 16 and mutations of E6 and E7 proteins stratified by cervical and oropharyngeal squamous cell carcinoma tissue, respectively. When comparing the difference of cervix and oropharynx tissues across HPV types or variants of HPV 16, the exact test along with the Boneferroni adjustment of multiple independent hypothesis tests was carried out. The Fisher exact test was employed to test the association between mutations of HPV16 E6 and E7 genes and tissue groups (cervix and oropharynx). Additionally, the exact trend test was used to assess the change in the percentage of HPV positive oropharyngeal squamous cell carcinoma samples from 2003 to 2016. All tests of statistical significance were two-sided with a P value < 0.05 indicating a significant difference. Analyses were performed using SAS version 9.4 (SAS Institute).

## Results

### Distribution of HPV types in cervical and oropharyngeal tumors

**Fig 1:**
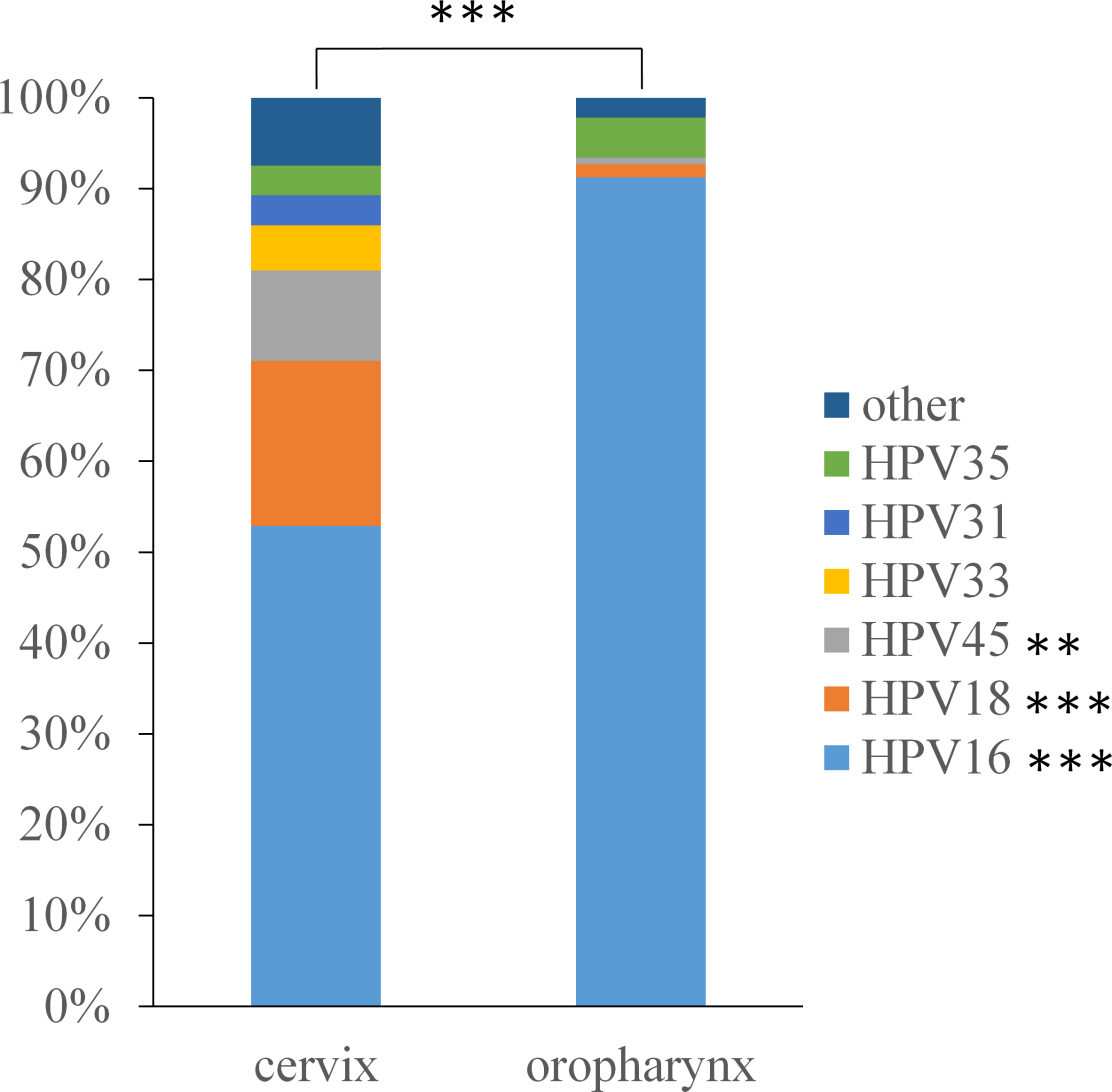
Distribution of HPV types. HPV type distribution found in oropharyngeal tumors differs significantly from the distribution in cervical cancer. HPV16 is more prevalent, while HPV18 and HPV45 are much less prevalent in the oropharynx than in the cervix.

Using the published GP5/GP6 PCR protocol [29] we established our HPV positive cancer sample sets of 137 oropharyngeal and 121 cervical tumors. Samples that did not produce positive GP5/GP6 PCR reactions were omitted from further studies. We found higher prevalence of HPV16, HPV35 and HPV67 in OSSC than in CSSC while all other HPV types we identified (18, 45, 33, 31, 56, 11, 30, 51, 53, 59, 69) showed lower prevalence in oropharynx (S1 Table). Three HPV types (16, 18, and 45) reached statistical significance when comparing prevalence in the oropharynx versus the cervix (Fig 1). HPV16 was present in the vast majority (91.2%) of oropharyngeal samples versus the much lower HPV16 ratio of 52.9% in the cervix (p<0.001). At the same time, HPV18 was present in 18.2% of HPV positive cervical samples versus 1.5% in the oropharynx (p<0.001); and HPV45 was present in 9.9% of HPV positive cervical samples versus 0.7% in the oropharynx (p = 0.006). A trend for higher HPV35 prevalence in oropharynx was also identified, but did not reach statistical significance. The overall distribution of HPV types found in the tumors of the oropharynx versus the cervix was significantly different (p<0.001).

### Distribution of HPV16 variants in cervical and oropharyngeal tumors

**Fig 2:**
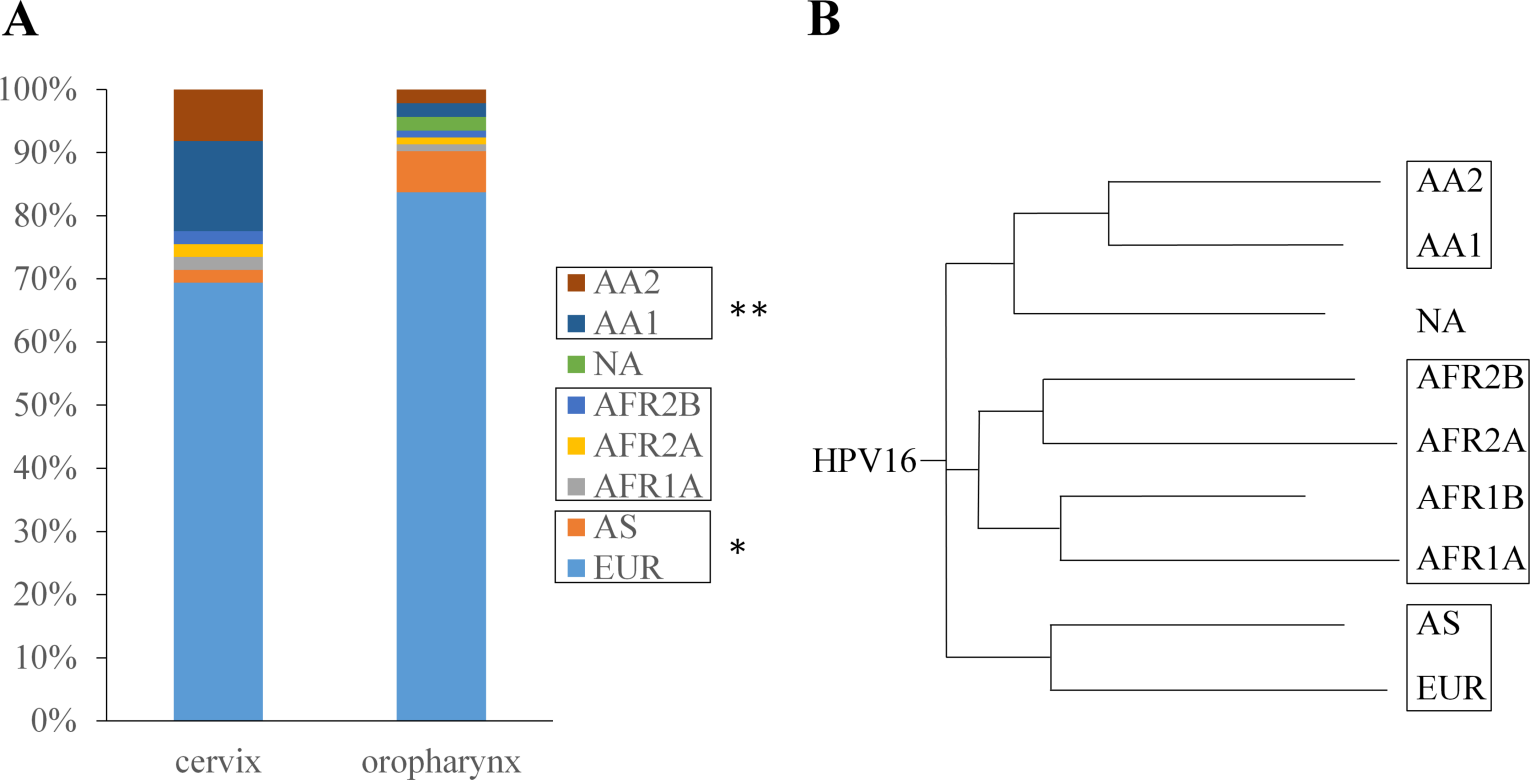
HPV16 variants. (A) The distributions of two HPV16 variant groups differ significantly in oropharyngeal and in cervical cancer. (B) Schematic phylogenetic tree for HPV16 based on Cornet et al. 2012 [30]. NA variants were considered a separate group. AA1, Asian American 1; AA2, Asian American 2; NA, North American; AFR1A, African 1A; AFR1B, African 1B; AFR2A, African 2A; AFR2B, African 2B; AS, Asian; EUR, European.

Using published PCR protocols [30] we identified the variant type of 92 HPV16 positive oropharyngeal samples and 49 HPV16 positive cervical samples (S2 Table). Based on these findings we compared the linage distribution of HPV16 positive samples from the cervix and the oropharynx (Fig 2A). Our samples included 8 variants: European (EUR), Asian (AS), African 1A (AFR1A), African 2A (AFR2A), African 2B (AFR2B), North American (NA), Asian American 1 (AA1), and Asian American 2 (AA2). Three variants were found at higher prevalence in oropharynx than in cervix (EUR, AS, NA), while the others were lower. The difference in prevalence for single variants in oropharyngeal versus cervical samples did not reach statistical significance, because of low sample size, but it is worth mentioning that the prevalence of AS and NA variants in oropharynx were multiple times higher than in cervix, while AA1 and AA2 variants were multiple times less prevalent (S2 Table).

Individual variant types were then combined into 4 groups based on phylogenic relation (Fig 2B) demonstrated by Cornet et al [30]: EUR+AS, AFR1A+AFR2A+AFR2B, NA, and AA1+AA2. When comparing the prevalence of these groups, two of them reached statistical significance (Fig 2A). The EUR+AS variant group showed higher prevalence in the oropharyngeal samples with 90.2% of all HPV16 positive samples belonging to this group, versus 71.4% of the cervical samples (p = 0.03). In contrast, the AA1+AA2 variant group was present in 22.5% of cervical samples versus only 4.4% of oropharyngeal samples (p = 0.01). A potential trend for higher NA prevalence in oropharynx was also noted, but the number of NA samples (2 in the oropharynx versus 0 in the cervix) were too low to reach statistical significance.

### Chronological trend in HPV-positive oropharyngeal cancer incidence

**Fig 3:**
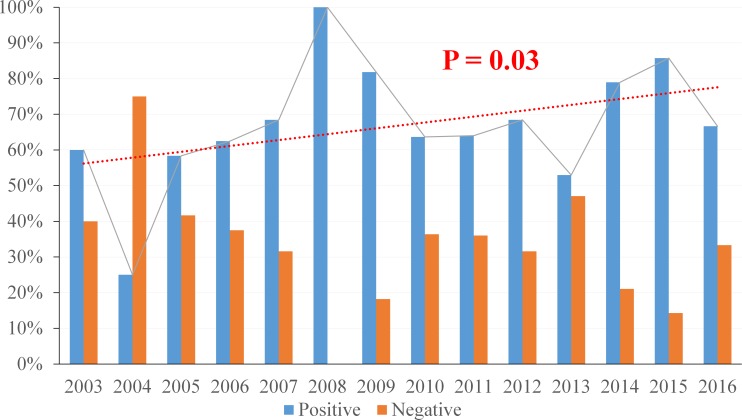
Percentage of HPV positive oropharyngeal squamous cell carcinomas by year. There is an increasing trend of HPV positive oropharyngeal squamous cell carcinomas over a 14 year period.

To determine the trend in the percentage of HPV positive OSCC (Fig 3), the data was analyzed with an exact trend test. From the years 2003 to 2016, there was an increasing trend in the percentage of HPV positive OSCC (p = 0.03), which corresponds to current literature. However, the numbers for years 2004 and 2008 could be considered outlier data and when these years were removed for reanalysis, the positive trend was no longer statistically significant (p = 0.27).

### Mutations in HPV16 E6 and E7 sequences

**Fig 4:**
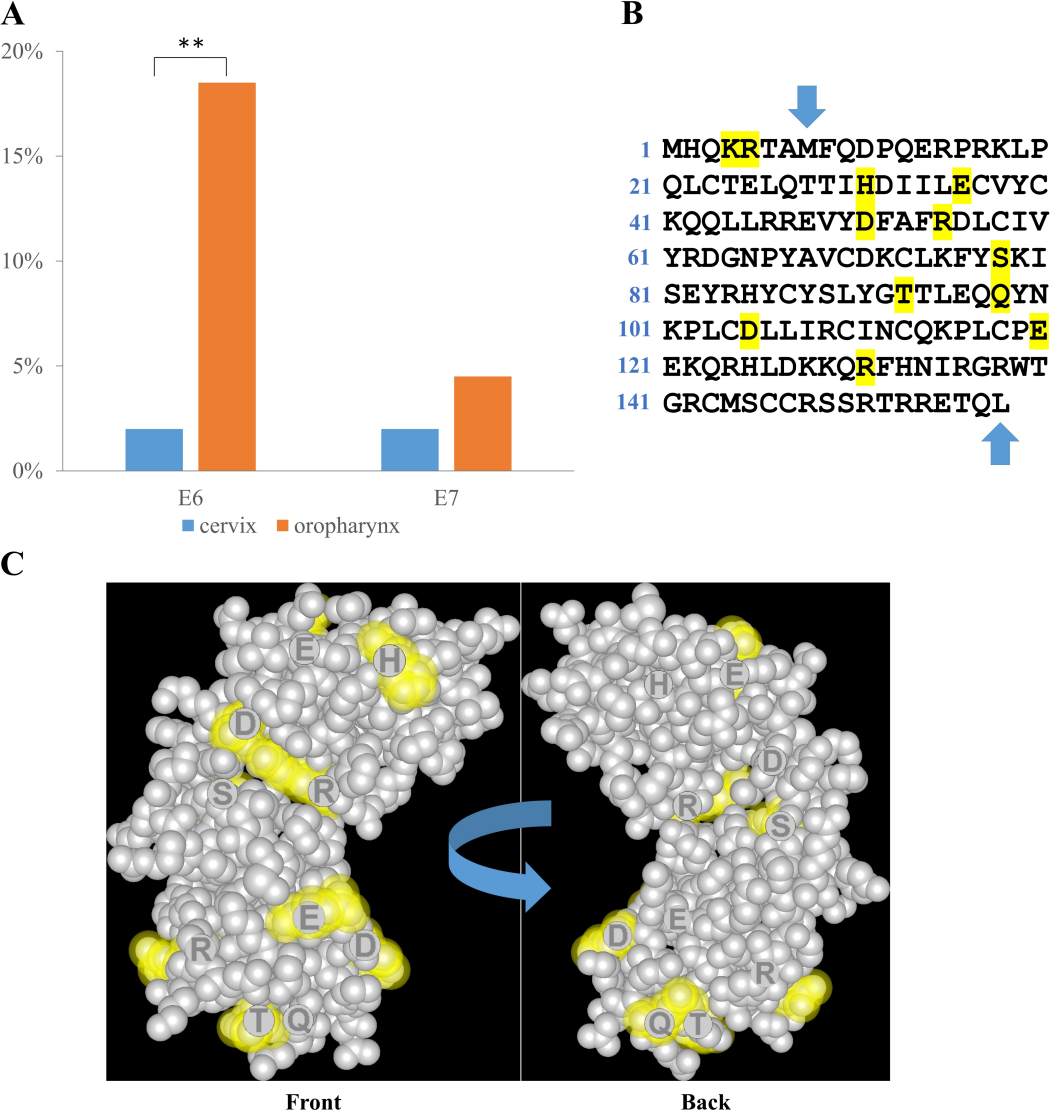
HPV16 mutations. (A) The percentage of HPV16 positive squamous cell carcinomas with mutant E6 protein is significantly higher in the oropharynx, than in the cervix. (B) Amino acid sequence of the HPV16 E6 protein. Mutation sites in OSCC are highlighted. Blue arrows mark the start and end points for the 3D model. (C) 3D structure of HPV16 E6 protein solved by Martinez-Zapien et al. 2016 [33]. The majority of the amino acids (yellow) replaced by mutations in the oropharynx are located on the surface.

The E6 and E7 genes from samples containing the HPV16 genome were sequenced and analyzed to identify mutations (Table 1) compared to the NCBI reference genome NC_001526.4. Since DNA extracted from FFPE tissue is often damaged we could only determine the complete sequence of the HPV16 E6 gene in 49 cervical and 92 oropharyngeal samples. Similarly, we were able to sequence the entire HPV16 E7 gene in 51 cervical and 89 oropharyngeal samples. We performed mutation analysis on these complete sequences only. When comparing the numbers of mutations for each protein between oropharynx and cervix (Fig 4A), previously reported polymorphisms (E6: 7392G, 7173G, 7151C) [17, 32] and silent mutations were excluded, only genomic mutations that resulted in amino acid change were considered.

**Table 1:** Mutations in HPV16 E6 and E7 genes and proteins.

Protein	Location	Samples*	Mutation	AA change	Sneath's index	Category
**E6**	**Oropharynx**		**New mutation**			
		1	7484C	E120D	7	conservative
		4	7357G	S78C	13	conservative
		1	7335A	D71N	14	conservative
		1	7417G	Q98R	23	semi-conservative
		1	7215A	H31N	24	semi-conservative
		1	7230A	E36K	26	semi-conservative
		1	7437T	D105Y	34	non-conservative
		1	7402A	T93K	34	non-conservative
		2	7135C/7287T	K4T/R55W	34/36	non-conservative
		1	7437C	D105H	35	non-conservative
		1	7516C	R131T	38	non-conservative
		1	7135A	R5G	43	non-conservative
		1	7137–7469	Deletion	NA	non-conservative
		1	7298T	none	NA	silent
		1	7496G	none	NA	silent
			**Polymorphism**			
		12	7392G	L90V	9	conservative
		4	7173G	R17G	43	non-conservative
		2	7151C	none	NA	silent
	**Cervix**		**New mutation**			
		1	7407G	L95V	9	conservative
		1	7229G	none	NA	silent
			**Polymorphism**			
		14	7392G	L90V	9	conservative
		1	7173G	R17G	43	non-conservative
		5	7151C	none	NA	silent
**E7**	**Oropharynx**		**New mutation**			
		1	7687C	L28F	19	semi-conservative
		2	7754A	H51N	24	semi-conservative
		1	7799T/7865C	R66W/G88R	36/43	non-conservative
		1	7864G	none	NA	silent
		1	7708G	none	NA	silent
		1	7720C	none	NA	silent
	**Cervix**		**New mutation**			
		1	7731A	G43E	37	non-conservative
		2	7864G	none	NA	silent

Mutations included in Fig 4 are shaded blue.

*Number of samples that contain the given mutation.

In the cervix, we only found 2 mutations: 1 out of 49 (1/49) samples presented E6 mutation and 1/51 showed E7 mutation. In the oropharynx these numbers were 17/92 for E6 and 4/89 for E7. Thus, when comparing anatomical locations, in the cervix 2.0% of the HPV16 samples had a mutation in the E6 gene versus 18.5% in the oropharynx, demonstrating a highly significant difference (p = 0.006). In contrast, for the E7 gene the mutation rate was 2.0% in the cervix and 4.5% in the oropharynx; this difference did not reach statistical significance (Fig 4A).

All mutations were found to be single nucleotide substitutions; except for one deletion of a 111 amino acid segment of E6 in one oropharyngeal sample. Most mutations were unique, occurring only once in our study. However, we found three reoccurring mutations, pinpointing potential hotspots: the substitution of serine78 to cysteine (S78C) in E6 that was found in four oropharyngeal samples, H51N in E7 occurring in two oropharyngeal samples, and the K4T/R55W pair of mutations in E6 that was identified in two oropharyngeal samples, interestingly, in the same pairing. In addition, it may be noteworthy, that aspartic acid 105 in E6 has been found altered in two different oropharyngeal samples, once to histidine (D105H) and once to tyrosine (D105Y).

The total number of OSCC samples harboring mutations was 21 (17 E6 and 4 E7 mutations), remarkably, these mutations suggested considerably high impact (non-conservative and semi-conservative mutations) on the structure and/or function of the protein (Table 1). In the oropharynx, 10/21 samples harbored non-conservative (SI:31–45) mutations, 6/21 samples had semi-conservative (SI:16–30) mutations, and only the mutations in 7/21 samples were conservative (SI:1–15). In the cervix the only E6 mutation we found was conservative, while the only E7 mutation was non-conservative.

Lastly, we analyzed the positions of mutations in the E6 protein in oropharyngeal samples (Fig 4B and 4C). The 3D protein structure model [33] did not include the first 7 amino acids as labeled on Fig 4B. Interestingly, 8 out of the 10 modeled mutation sites are located on the surface of the E6 molecule (Fig 4C).

## Discussion

While cervical cancer has become the paradigm of HPV-associated cancer with extensively researched epidemiology and pathomechanisms, established screening and vaccination protocols, most of the characteristics of HPV-associated oropharyngeal cancer are much less understood. In this study, we compared genomic aspects of HPV associated with cervical versus oropharyngeal tumors in a geographically defined cohort of patients. To our knowledge this is the first report of the kind.

We showed significant differences in HPV type distribution between cancers of the cervix and the oropharynx. We found HPV16 to be more prevalent, while HPV18 and HPV45 much less prevalent in OSCC than in CSCC. In the cervix HPV16 showed over 50% prevalence, and together with HPV18 these two types were present in over 70% of all HPV-positive cancers. HPV45 contributed another 10%, followed by 11 additional HPV types with less than 5% prevalence each. These results are consistent with other reports including recent large studies [4, 7]. For HPV-positive OSCC we found HPV16 with over 90% prevalence while HPV18 and HPV45 presented at less than 2% each. All other HPV types were at very low prevalence (less than 2%) or completely absent, with the surprising exception of HPV35 that was present in over 4% of our samples. While all of our other results for HPV type distribution in OSCC are in line with available literature [9, 34, 35], HPV35 being the second largest contributor to oropharyngeal carcinogenesis is a unique finding of this study. Although the overall sample size was low (6 out of 137), further investigation for the reasons of this relatively high HPV35 prevalence could be warranted. In general, the significant overall difference in HPV type distribution between cancers of the two anatomical sites supports the notion that the epidemiology and pathomechanisms of HPV-driven carcinogenesis in the oropharynx may be different from those of the well-studied cervical cancer.

HPV16 is by far the most prevalent HPV type associated with both cervical and oropharyngeal cancer worldwide [4, 9]. Early studies identified six phylogenic clusters of HPV16 genomic variants, based on their original geographic distribution: African 1, African 2, Asian, Asian American, European and North American [36, 37]. Subsequent studies identified subclasses within the original variants based on specific nucleotide changes present in the E6, L1, L2 and LCR genes [30, 38]. Genetic differences between HPV16 variants have been suggested to account for altered tumorigenic risk [14, 15]. Despite its potential importance, very little data has been published about HPV16 variants distribution in cervical cancer [30, 38, 39] and virtually none for OSCC. We described here the HPV16 variants distribution of both CSCC and OSCC samples of our cohort, and compared the two sets with each other. When comparing individual HPV16 variant distributions, we only found subtle differences between cervical and oropharyngeal cancer samples, but we observed trends for higher AS and NA and lower AA1 and AA2 prevalence in OSCC than in CSCC–none of which reached the level of significance. This result may be unexpected after finding much greater differences in HPV type distribution, but since both anatomical sites were predominantly associated with the EUR variant (70% in CSCC and 83% in OSCC), all other variants were present in too low numbers for significant differences. For this reason we re-evaluated our data after combining individual HPV16 variants into 4 groups based on phylogenic relation [30]. When comparing the prevalence of these groups, in oropharyngeal samples the EUR+AS variant group showed significantly higher prevalence (90.2% versus 71.4%) and the AA1+AA2 variant group showed significantly lower prevalence (4.4% versus 22.5%) than in cervical samples. Although NA variants should be grouped with the AA variants based on genetic similarities [30] we decided to keep them as a separate group because they were showing on opposite trend (higher prevalence in OSCC than in CSCC). This finding–along with the differences between phylogenic groups–may lead to a better understanding of the differences in HPV-driven oncogenic pathomechanisms between the oropharynx and the cervix, once the corresponding structural and functional differences between these variant groups are elucidated in further studies. Our results for HPV-type and HPV16 variant distribution in the oropharynx could have implications regarding vaccine design and therapies.

Recent studies have shown that HPV prevalence in OSCC increased dramatically in the past decades from less than 20% to the currently reported levels of over 70% in the United States [2, 20]. When we examined HPV prevalence in oropharyngeal cancers over calendar time, we also found an increasing trend in HPV-positive OSCC rates, but this trend was not very robust. In fact, the increase became statistically non-significant when we left out the highest and lowest data points (2004, 2008). While our trendline showed an increase from approximately 60% prevalence in 2003 to around 80% in 2016, most of the available literature suggests steeper increase rates [20, 40]. This discrepancy may originate from the relatively small and uneven yearly sample size of this study. Another possible explanation is that according to some reports, most of the rapid increase in prevalence took place between 1984 and 2004 [20], and current prevalence numbers in the US are approaching a plateau. Larger studies will be needed to better clarify this trend.

HPV phylogenic variants differ from each other by less than 10% of the genome [41] and variant-dependent tumorigenic risk have been recently proposed, as discussed above, but not much is known about the effect of single mutations in the viral genome on oncogenic potential. A few recent studies assessed single mutation profiles of the E6 and E7 genes in cervical cancer [42–44] but no similar data exist in the literature for HPV-associated oropharyngeal cancer. In this study we reported all previously unknown mutations found in CSCC and OSCC samples compared to the NCBI reference genome of HPV16 E6 and E7 genes; and demonstrated that while there was a much higher mutation rate of the E6 gene in OSCC (18.5%) than in CSCC (2.0%), the E7 gene in both locations was conserved. This finding is in agreement with recent cervical cancer studies [42–44], and extends the apparent restriction on E7 mutations to the oropharynx as well. While this may imply that E7 is the most vital gene for HPV-driven oncogenesis, E6 may be equally important, but more flexible in maintaining its functions despite some mutations. The fact that we did not find any frameshift mutations in E6 supports this latter explanation. According to this view, with the higher number of new mutations the virus would be adjusting to the new anatomical environment of the oropharynx resulting in an emerging disease, while in the cervix HPV-driven cancer would be much more settled. Nevertheless, the invariability of E7 presents an attractive potential target for therapy at both locations.

Assessment of the structural and functional implications of all new mutations we found is beyond the scope of this study, but it is worth mentioning that reoccurring mutations may pinpoint potential mutational hotspots in OSCC (S78C in E6 and H51N in E7), while the fact that K4T and R55W in E6 was found together in two different OSCC samples may suggest that their effects could complement each other. Aspartic acid 105 in E6 may be another potential hotspot that has not “adopted” its optimal amino acid yet for OSCC, since we found it substituted in two different samples but not the same way (D105H and D105Y).

3D modeling of the E6 protein revealed that the majority of the mutation sites (80%) in the oropharynx are located on the surface of the molecule. These mutations could potentially alter E6 interactions with host proteins in the tumor cell, and/or they could be accompanied by corresponding mutations in the interacting host cell proteins. In search of such corresponding mutations, we analyzed the genomic DNA sequences of the p53 protein in the OSCC samples harboring E6 mutations, but all these p53 sequences were found to be wild type (data not shown). Further investigation into the role of these mutations in E6 functions will be needed.

One puzzling result of this study is the high rate of non-conservative and semi-conservative mutations (70% altogether) found in OSCC. One possible explanation could be (along the lines of the earlier proposed reason for high E6 mutations rates) that the oropharyngeal environment is exerting a selective pressure on HPV16 to rapidly adapt to new physiological conditions [45]. If this is true, HPV may present an even greater threat in the near future with enhanced infectivity/replication/oncogenic capabilities in the oropharynx.

## Conclusion

Our study, using DNA sequence analysis, describes significant genomic differences between the HPV genomes associated with cervical and oropharyngeal cancers diagnosed in a Southeast Texas community. We showed that cancers of the two anatomical locations differ in HPV type and variant distribution as well as mutation frequencies of the viral E6 gene. Our study also provided evidence that, as opposed to the E6 gene, the E7 gene is genetically conserved in cancers of both the cervix and the oropharynx. Our data suggests that while HPV-associated OSCC and CSCC share common biological features, they present important differences in HPV-genome that may explain their distinct pathomechanisms and susceptibility to treatment. New screening programs, vaccination and treatment strategies, specifically designed for OSCC, will be necessary to reduce the burden of this emerging disease. To achieve these goals, further studies will be needed to understand the specific pathobiology of HPV-driven oncogenesis in the oropharynx.

## Supporting information

S1 Table Distribution of HPV types. (XLSX)

S2 Table Distribution of HPV 16 variants. (XLSX)
